# Supplementary material for: Enzymatic Characterization of the Isocitrate Dehydrogenase with Dual Coenzyme Specificity from the Marine Bacterium Umbonibacter marinipuiceus
Source: Int J Mol Sci. 2023 Jul 13;24(14):11428. doi: 10.3390/ijms241411428 (PMC10380307; doi:10.3390/ijms241411428)
Supplement: Supplementary file 1 [file ijms-24-11428-s001.zip › ijms-2504730-supplementary.pdf]

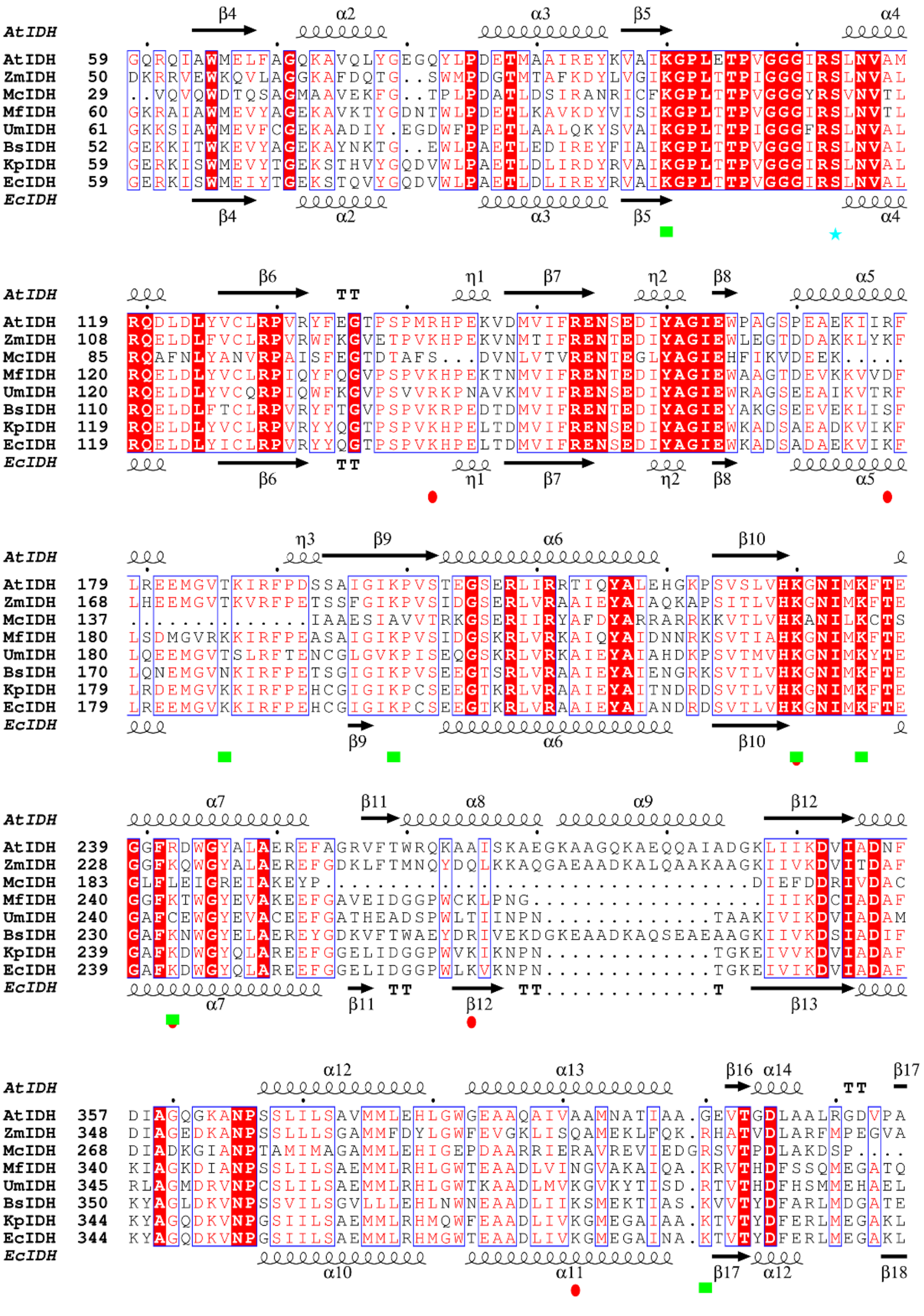

Figure S1. Structure-based amino acid sequence alignment. The comparison of UmIDH with AtIDH, ZmIDH, McIDH, EcIDH, BsIDH, KpIDH, and dual coenzyme dependent MfIDH. The structure of AtIDH (PDB ID: 2D4V) and EcIDH (PDB ID: 3ICD) were downloaded from the PDB database. The conserved phosphorylation sites were indicated by cyan star. The lysine residues that may be acetylated or succinylated in EcIDH were indicated by red circles and green squares, respectively. The figure was created by ESPript 3.0.
